# Effect of Fiber Type and Content on Surface Quality and Removal Mechanism of Fiber-Reinforced Polyetheretherketone in Ultra-Precision Grinding

**DOI:** 10.3390/polym14194223

**Published:** 2022-10-08

**Authors:** Shang Gao, Xinyu Zhou, Jiani Guo, Renke Kang

**Affiliations:** Key Laboratory for Precision and Non-Traditional Machining Technology of Ministry of Education, Dalian University of Technology, Dalian 116024, China

**Keywords:** polyetheretherketone, fiber-reinforced PEEK, surface roughness, surface morphology, material removal mechanism

## Abstract

Polyetheretherketone (PEEK) is a promising thermo-plastic polymer material due to its excellent mechanical properties. To further improve the mechanical properties of PEEK, different kinds of short fibers are added into the PEEK matrix. The grinding machinability of short-fiber-reinforced PEEK varies with the effect of fiber type and content. Therefore, it is crucial to investigate the surface quality and removal mechanism of fiber-reinforced PEEK in ultra-precision grinding. In this paper, different fiber types and mass fractions of short-fiber-reinforced PEEK, including carbon-fiber-reinforced PEEK (CF/PEEK) and glass-fiber-reinforced PEEK (GF/PEEK), are employed. The grinding machinability of short-fiber-reinforced PEEK was investigated using grinding experiments with grinding wheels of different grit sizes. The effects of the fiber type and mass fraction on the surface quality and removal mechanism during grinding were discussed. The results showed that the brittle–ductile transition depth of carbon fiber was much larger than that of glass fiber, so it was easier to achieve ductile removal in grinding with the carbon fiber. Therefore, the ground surface roughness of CF/PEEK was smaller than that of GF/PEEK under the same grinding conditions. With the increase in carbon fiber mass fraction, the ground surface roughness of CF/PEEK decreased due to the higher hardness. The brittle–ductile transition depth of glass fiber was small, and it was easy to achieve brittle removal when grinding. When the glass fiber removal mode was brittle removal, the GF/PEEK surface roughness increased with the increase in glass fiber content.

## 1. Introduction

Polyetheretherketone (PEEK) is a new kind of thermo-plastic polymer material, which has a low density, high mechanical strength, good electrical insulation performance, corrosion resistance, self-lubrication performance and a series of other excellent properties. It is widely used in aerospace, automotive and other fields [1,2,3], and especially in the medical field as medical implants [4,5]. However, PEEK has some disadvantages, such as a small elastic modulus that cannot meet the needs of some fields. By adding different types and mass fractions of dispersed fibers or nano-reinforced particles to a material’s matrix, this common method can enhance its mechanical properties [6,7,8,9,10,11]. Due to carbon fiber and glass fiber having the advantages of high strength, high modulus and small thermal expansion coefficient, they are widely used as reinforcements to improve the physical properties of polymer materials [12,13]. Therefore, by adding carbon fiber or glass fiber to PEEK, researchers created a carbon-fiber-reinforced PEEK composite (CF/PEEK) or glass-fiber-reinforced PEEK composite (GF/PEEK) [14], which further improved the mechanical and tribological properties of the material [15], thus, expanding the application range of PEEK. In actual production, series of mechanical processes are usually required to meet the dimensional accuracy and surface quality requirements of a product. Therefore, studying the machinability of PEEK and fiber-reinforced PEEK has important reference value for the processing of related products.

Current research on the machining of PEEK and fiber-reinforced PEEK has mainly focused on turning, milling, grinding and lapping. Ji et al. [16] investigated the turning performance of PEEK and glass-fiber-reinforced PEEK, and compared the PV values and roughness values (Ra) of the two materials after machining. The results showed that the PV and roughness values (Ra) of glass-reinforced PEEK were greater than pure PEEK, and glass-reinforced PEEK had a poor turning performance. Davim et al. [17] also studied the turning properties of PEEK and glass-fiber-reinforced PEEK. The results showed that the surface roughness of the material decreased with the increase in cutting speed, and the surface roughness of pure PEEK was lower than that of glass-fiber-reinforced PEEK under the same machining parameters. Yan et al. [18] studied the milling of three thermo-plastic polymer materials, including PEEK, PI and PMMA. The results showed that as the temperature of the machining area increased, the material entered a visco-elastic state and the quality of the milled surface deteriorated. Khoran et al. [19,20] studied the grinding performance of PEEK, carbon-fiber-reinforced PEEK and glass-fiber-reinforced PEEK. The results showed that the machining temperature had a greater impact on the surface quality of PEEK and fiber-reinforced PEEK, while when using liquid nitrogen as the coolant, the grinding surface quality was significantly improved. Under the same processing parameters, the surface roughness of carbon-fiber-reinforced PEEK was the smallest, and that of pure PEEK was the largest. Because the surface quality of PEEK and fiber-reinforced PEEK is temperature sensitive, the surface roughness of each material tends to decrease first and then increase with the increase in cutting speed. Gao et al. [21] studied the lapping performance of PEEK, carbon-fiber-reinforced PEEK and glass-fiber-reinforced PEEK. The results showed that the surface roughness and material removal rate of fiber-reinforced PEEK were lower than those of pure PEEK under the same grinding parameters.

In the ultra-precision machining process, grinding is generally regarded as a component of the finishing process, and the dimensional accuracy and surface quality of parts are often affected by the grinding process. At present, little research has been conducted on the effect of fiber type and content on the grinding surface roughness and grinding surface morphology of fiber-reinforced PEEK, and the material removal mechanism of fiber-reinforced PEEK in grinding has not been revealed. As a consequence, in this paper, the surface morphology and roughness of PEEK reinforced with different fiber types and different fiber mass fractions were analyzed. The material removal mechanism of fiber reinforcement was revealed by calculating the grit cutting depth, and the reasons for the differences in roughness after grinding were explained based on the changes of mechanical properties of various materials.

## 2. Materials and Methods

### 2.1. Materials

**Figure 1:**
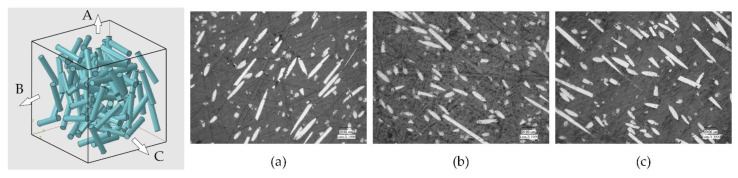
Microstructure photographs of the sample in three different directions, taking CF30/PEEK as an example: (**a**) A direction (x-z plane); (**b**) B direction (y-z plane); (**c**) C direction (x-y plane).

The materials used in the experiments were purchased from Nanjing Shousu Special Engineering Plastic Products Co, Ltd. (Nanjing, China). The experiment included five materials, pure PEEK, 10% carbon-fiber-reinforced PEEK (CF10/PEEK), 30% carbon-fiber-reinforced PEEK (CF30/PEEK), 10% glass-fiber-reinforced PEEK (GF10/PEEK) and 30% glass-fiber-reinforced PEEK (GF30/PEEK). The carbon fiber was PAN-based short carbon fiber and the glass fiber was E-glass short fiber. The diameter and length of the carbon fiber and glass fiber followed a normal distribution. The average diameter and length of the carbon fiber were 8 and 40 μm, respectively. The average diameter and length of the glass fiber were 10 and 60 μm, respectively. Taking the CF30/PEEK material as an example, the microstructure photograph of the sample is shown in Figure 1, where the fibers were randomly distributed in the PEEK matrix. All materials were produced into plates through an extrusion process and then machined into 15 mm × 15 mm × 3.5 mm samples using a wire cutting machine. In order to ensure the flatness of the workpiece surface and to reduce the influence on the experimental results, the material’s surface was abraded with #400 silicon carbide abrasive paper. The mechanical properties of the five materials used in the experiments are shown in Table 1.

### 2.2. Methods

**Figure 2:**
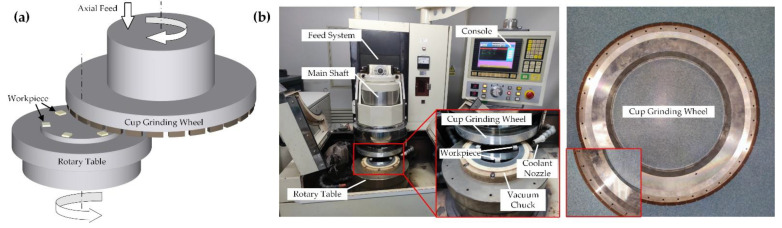
Grinding experimental setup: (**a**) schematic view of workpiece rotational grinding; (**b**) VG401 MK II workpiece rotational grinding machine and cup grinding wheel.

All grinding experiments were carried out on a workpiece rotational ultra-precision grinding machine (VG40 MK II). Before grinding, the sample was attached to the rotary table. In grinding, the grinding wheel type was a resin-bonded diamond wheel with de-ionized water as the coolant. The schematic diagram of the workpiece rotational grinding and the machine tool equipment used for the grinding experiment are shown in Figure 2.

In the grinding process, besides the grit size of the grinding wheel, the grinding parameters also had great influence on the surface roughness of the material. In this study, grinding experiments with different grinding parameters of pure PEEK materials were first carried out with a #325 grinding wheel to explore the influence of the grinding parameters on the surface roughness of the materials; the experimental parameters are listed in Table 2. In addition, we used three different grit sizes of grinding wheel for the grinding experiments, including #325, #600 and #2000 grinding wheels, to explore different grit sizes of grinding wheels on the influence of different surface roughnesses, and to compare the surface roughness of different fiber-reinforced PEEK under the same grinding conditions; the experimental parameters were set as that listed in Table 3.

The analysis of the material grinding surface roughness and surface morphology is an important method for exploring the quality of material grinding surface processing. In this experiment, Taylor profiler instruments (CLI2000, Taylor Hobson Ltd., Leicester, UK) were used to measure the sample surface roughness. Five different locations on the ground surface were measured to ensure the reliability of the roughness value, and the average was taken as the final result. The surface morphology was observed using a scanning electron microscope (FEI-Q45, US).

## 3. Results

### 3.1. Surface Roughness of Pure PEEK and Fiber-Reinforced PEEK

**Figure 3:**
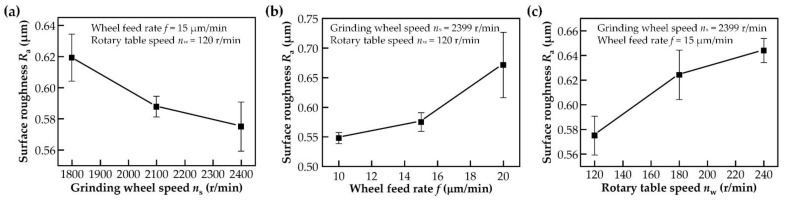
Effect of different grinding parameters on the surface roughness of PEEK: (**a**) effect of grinding wheel speed; (**b**) effect of wheel feed rate; (**c**) effect of rotary table speed.

The analysis of the surface roughness of a workpiece is an important method for evaluating surface quality. Figure 3 shows the effects of the grinding wheel speed, grinding wheel feed rate and table speed on the surface roughness of PEEK. It can be seen that the grinding surface roughness of PEEK had a significant reduction with the increase in grinding wheel speed and decrease in grinding wheel feed rate and table speed.

**Figure 4:**
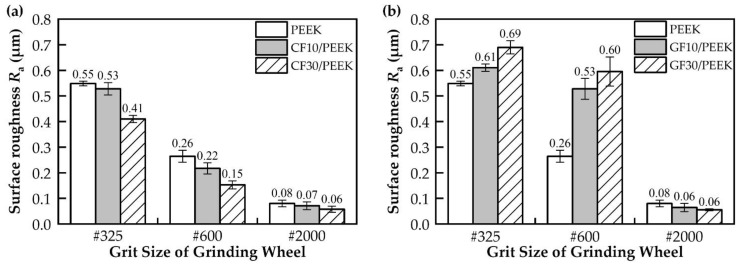
Comparison of surface roughness results for different fiber mass fractions: (**a**) the surface roughness of the pure PEEK, CF10/PEEK and CF30/PEEK; (**b**) the surface roughness of the pure PEEK, GF10/PEEK and GF30/PEEK.

**Figure 5:**
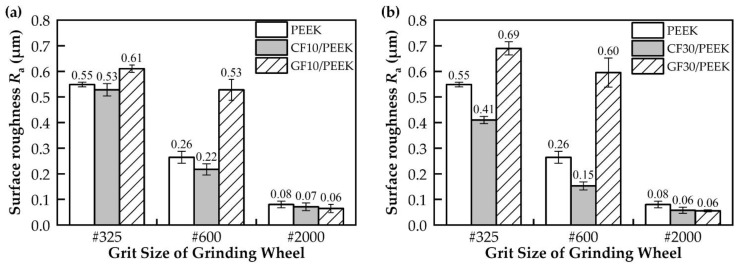
Comparison of surface roughness results for different fiber types: (**a**) the surface roughness of the pure PEEK, CF10/PEEK and GF10/PEEK; (**b**) the surface roughness of the pure PEEK, CF30/PEEK and GF30/PEEK.

The results of the analysis of the surface roughness with different grit sizes of grinding wheel are shown in Figure 4 and Figure 5. The surface roughness of PEEK and fiber-reinforced PEEK tended to decrease with an increasing grit size of the mesh (#325, #600 and #2000). Figure 4 shows the effect of the fiber mass fraction on the surface roughness of fiber-reinforced PEEK. As seen in Figure 4a, the surface roughness of CF30/PEEK was lower than CF10/PEEK under the same grinding conditions. The surface roughness of carbon-fiber-reinforced PEEK decreased with the increasing carbon fiber mass fraction. Figure 4b shows the grinding surface roughness results for PEEK reinforced with different glass fiber mass fractions. It can be seen that the changing trend in the roughness of glass-fiber-reinforced PEEK was significantly different from that of carbon-fiber-reinforced PEEK. GF30/PEEK had a larger surface roughness than GF10/PEEK under #325 and #600 wheel grinding conditions, while GF30/PEEK and GF10/PEEK had a similar surface roughness under #2000 wheel grinding conditions.

Figure 5 shows the effect of the fiber type on the surface roughness of fiber-reinforced PEEK. It can be seen that the surface roughness of carbon-fiber-reinforced PEEK was smaller than glass-fiber-reinforced PEEK under #325 and #600 wheel grinding conditions, while the surface roughness of carbon-fiber-reinforced PEEK and glass-fiber-reinforced PEEK was similar under #2000 wheel grinding conditions. CF/PEEK had a better grinding machinability than GF/PEEK.

### 3.2. Surface Morphology of Pure PEEK and Fiber-Reinforced PEEK

The observation of the grinding surface morphology was an important means to analyze the material removal mechanism and the form of material surface damage during the grinding process.

**Figure 6:**
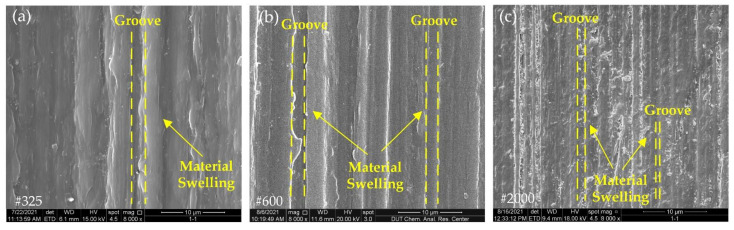
Surface morphology of PEEK with different grinding wheels: (**a**) grit size #325; (**b**) grit size #600; (**c**) grit size #2000.

Figure 6 shows the surface morphology of pure PEEK after grinding. The PEEK grinding surface had a lot of grinding grooves, and there was obvious material swelling on both sides of the grooves. Since the #2000 wheel had a smaller abrasive grit size, the number of grits per unit volume increased. As seen in Figure 6c, the width of the grooves on the grinding surface became narrower and the material swelling on both sides of the grooves reduced further.

**Figure 7:**
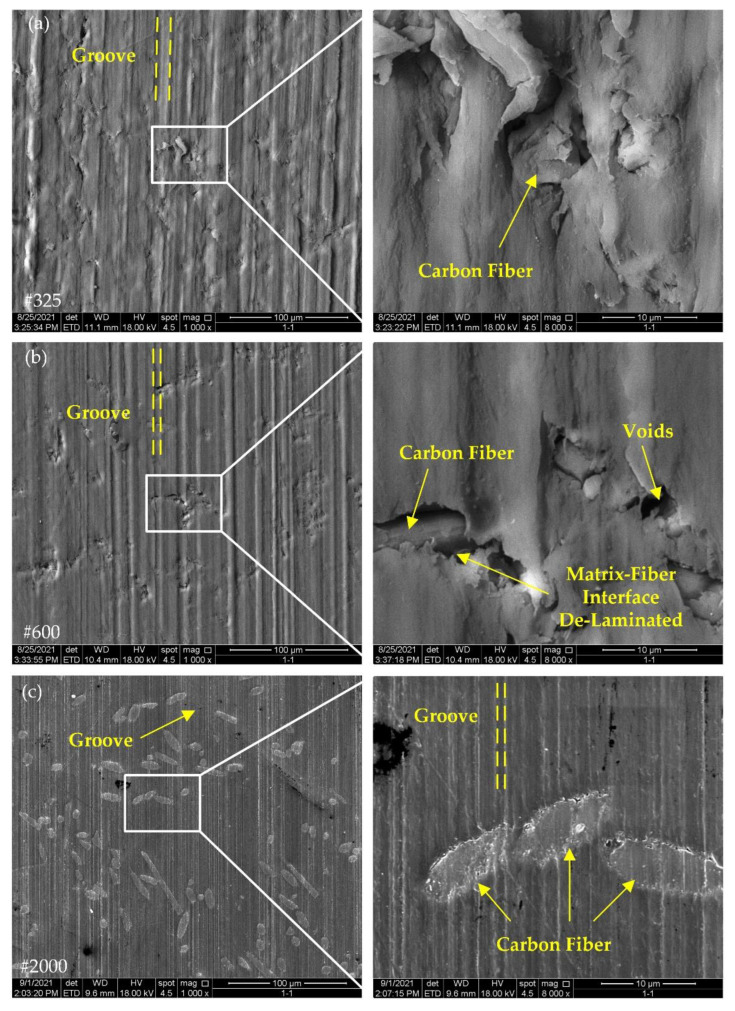
Surface morphology of CF10/PEEK with different grinding wheels: (**a**) grit size #325; (**b**) grit size #600; (**c**) grit size #2000.

**Figure 8:**
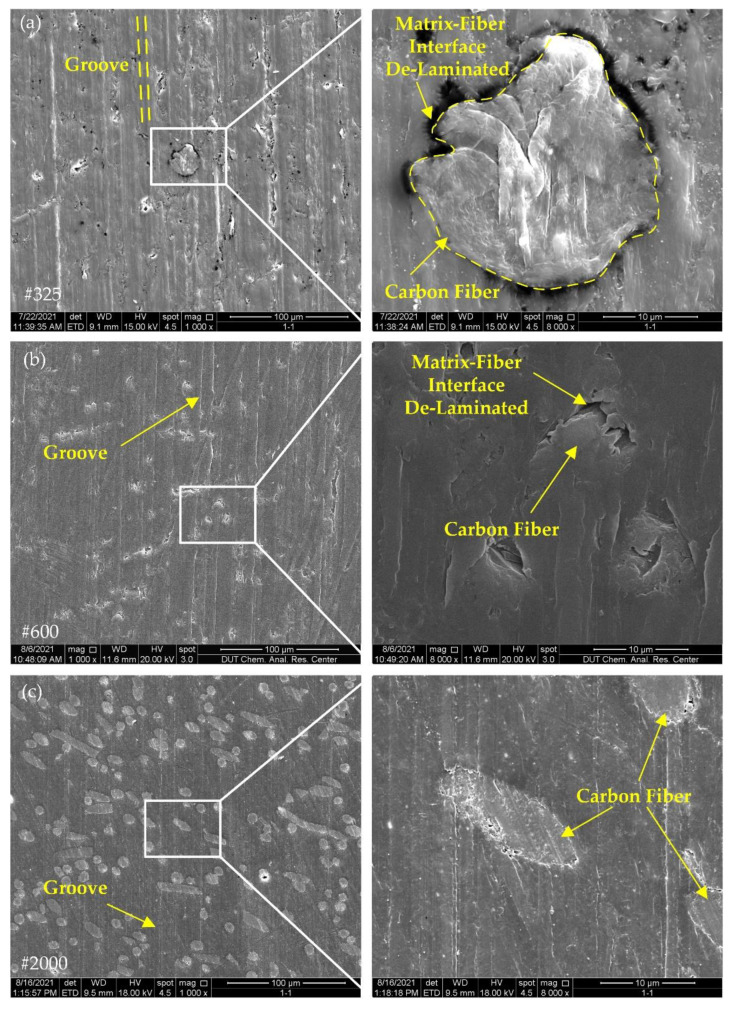
Surface morphology of CF30/PEEK with different grinding wheels: (**a**) grit size #325; (**b**) grit size #600; (**c**) grit size #2000.

Figure 7 and Figure 8 show the surface morphology of the 10% and 30% carbon-fiber-reinforced PEEK, respectively. As the size of grits in the #325, #600 and #2000 grinding wheels gradually became smaller, the width of a single groove also became smaller. On the other hand, due to the size of the grits inside the #325 and #600 wheels being large, the dispersion of the grits inside the wheels was relatively large and the height of grits protruding from the surface layer of the grinding wheel was less consistent. Therefore, in Figure 7a,b, some of the PEEK matrix showed a protruding shape, some had grinding grooves and the peak and valley positions of the surface profile were spread over a large distance. The size of the grit inside the #2000 grinding wheel was small, with an average radius of only 3.25 μm, and the height of the grit protruding from the surface layer of the grinding wheel decreased. Therefore, with the gradual reduction in the grit size, the grooves gradually tended to become denser and the height consistency between the grooves was improved, which also indicated that the surface quality could be significantly improved by grinding with the #2000 wheel.

Clear grooves could be seen in Figure 7a, while in Figure 8a, the surface grooves were less visible and the surface quality was improved to a greater extent. It can be seen that the change in carbon fiber content had an effect on the surface morphology of the material after grinding, and the increase in carbon fiber content could enhance the surface quality of PEEK after machining. In Figure 7b and Figure 8b, a similar regularity could be derived by comparing the differences in grooves between the two figures. In Figure 7c and Figure 8c, the two materials had a more similar morphology, and the effect of carbon fiber content on the surface quality gradually diminished.

Fiber–matrix de-bonding is one of the basic forms of damage in composites. In Figure 7a,b and Figure 8a,b, fiber–matrix de-bonding was generated at the interface location between the PEEK matrix and carbon fibers. Due to the high plasticity of the PEEK matrix, the plastic flowing PEEK could cover the fibers inside the material during the grinding process. The carbon fiber content did not appear to have a large effect on the fiber–matrix interface damage. After grinding with the #2000 grinding wheel, the surface quality was higher, the damage at the interface between the carbon fibers and the matrix had improved considerably and the internal reinforcing fibers were clearly seen on the surface.

**Figure 9:**
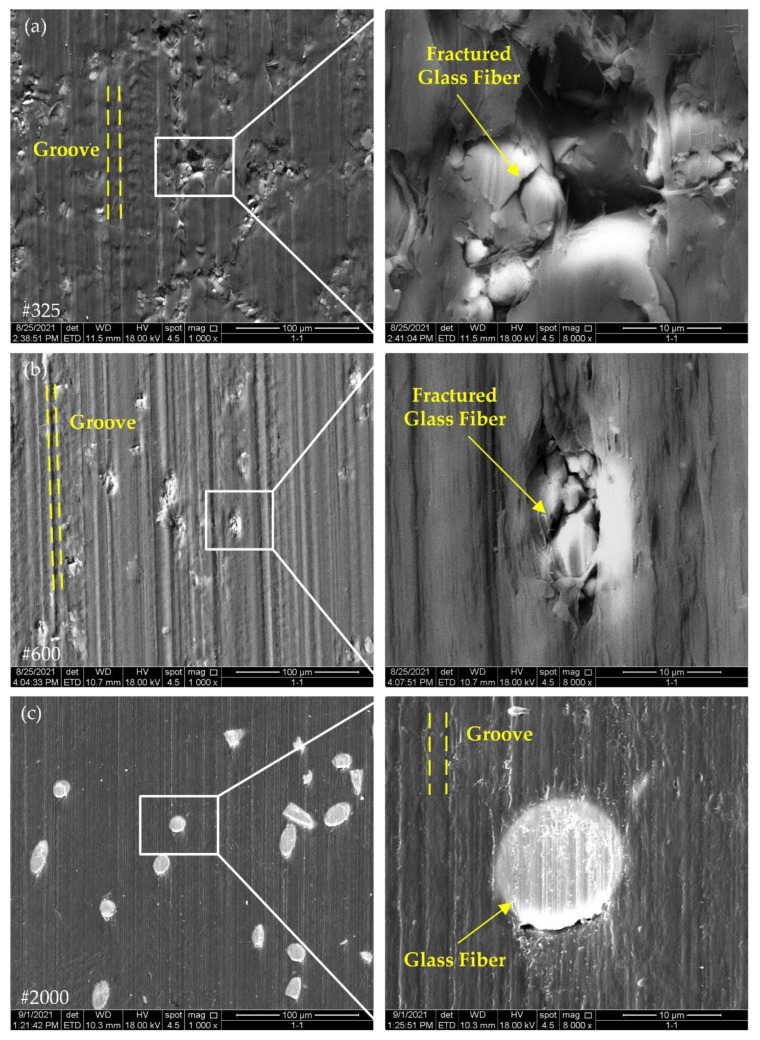
Surface morphology of GF10/PEEK with different grinding wheels: (**a**) grit size #325; (**b**) grit size #600; (**c**) grit size #2000.

**Figure 10:**
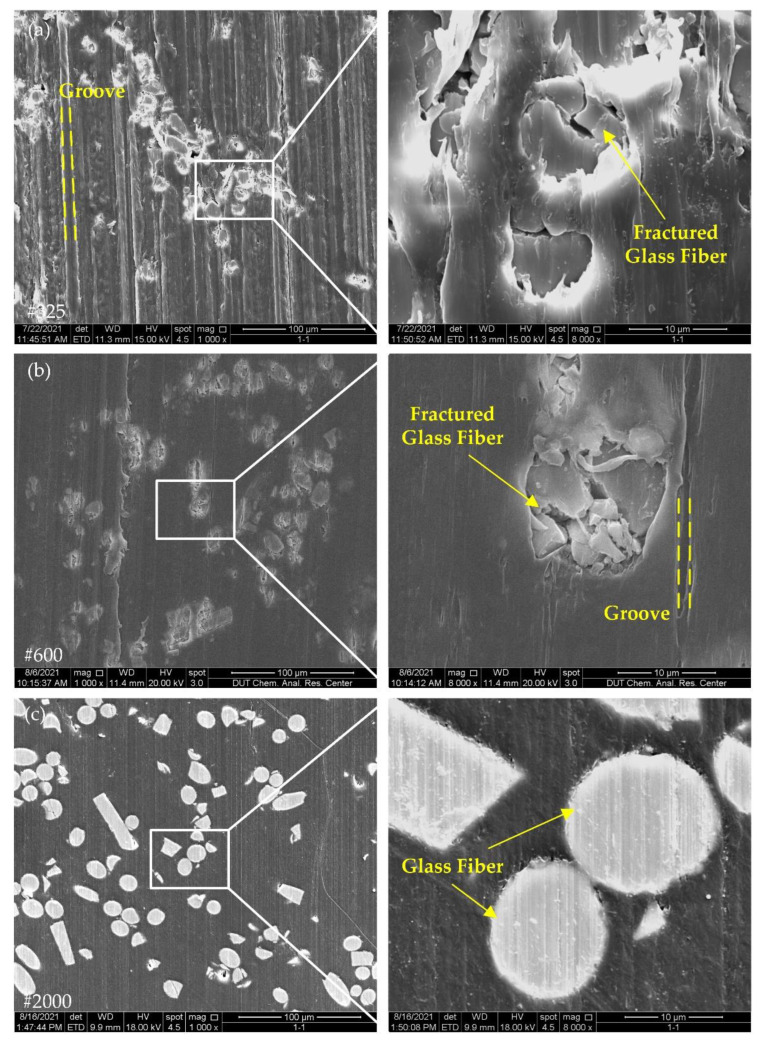
Surface morphology of GF30/PEEK with different grinding wheels: (**a**) grit size #325; (**b**) grit size #600; (**c**) grit size #2000.

Figure 9 and Figure 10 show the surface morphology of glass-fiber-reinforced PEEK. With the change in grit size, the groove pattern of GF/PEEK was similar to that of CF/PEEK, and the grooves after grinding with #325 and #600 wheels were dramatically different from the #2000 wheels. The internal size of the grit in the #2000 wheel was small, and the height of the grit protruding from the surface layer of the wheel was also smaller, so the grooves in Figure 9c and Figure 10c were more dense.

In Figure 9a,b and Figure 10a,b, due to the high brittleness of the glass fibers, there was considerable damage on the surface of the glass fiber reinforcement, and many cracks appeared on the surface of the glass fibers, which was very different from the removal pattern of the carbon fiber reinforcement. With the increase in glass fiber content, in Figure 10, more broken glass fibers could be observed on the surface, which reduced the surface quality of the material. In Figure 9c and Figure 10c, only small grooved were present on the glass fiber surface, proving that the removal mode of the glass fibers varied with the grinding wheel grit.

## 4. Discussion

### 4.1. Mechanism for the Creation of Surface Morphology

Both carbon-fiber-reinforced PEEK and glass-fiber-reinforced PEEK contained a PEEK matrix and fiber reinforcement, so the formation mechanism of their surface morphology was also determined with the removal mechanism of the two materials.

PEEK is a plastic material. During the grinding process, as the grit continued to penetrate into the workpiece surface, the plastic material went through three different stages, namely, friction, plowing and cutting [23], and, finally, formed chips, and material swelling appeared on both sides of the grinding grooves. In the surface morphology results of the fiber-reinforced PEEK composites in Figure 7, Figure 8, Figure 9 and Figure 10, clear grinding grooves could be observed in all PEEK parts, and material swelling appeared on both sides of the grinding grooves, which indicated that the removal mechanism of the PEEK part was ductile removal.

Carbon fiber and glass fiber belong to brittle materials [24,25]. The removal mechanism of brittle materials can be divided into brittle removal and ductile removal. When the material is removed with brittle removal, a large number of cracks can appear on the surface of the material; when the material is removed with ductile removal, the surface of the material can show ductile removal marks similar to those of plastic materials [26]. In Figure 9 and Figure 10, the glass fiber surfaces ground with the #325 and #600 grinding wheels showed obvious cracks, while the glass fibers were more intact after grinding with the #2000 grinding wheels. It follows that the removal mechanism of the glass fibers changed with grinding wheels of different grit sizes.

In the study about the removal mechanism of brittle materials, based on the removal energy, Bifano [27] proposed a classical critical depth of cut model for the brittle–ductile transformation of hard and brittle materials, as shown in Equation (1):

(1) $$d_{c}=\beta \left(\frac{E}{H}\right){\left(\frac{K_{c}}{H}\right)}^{2}$$

where *β* is the integrated coefficient of the model, *E*, *H* and *K*_*c*_ are the elastic modulus, hardness and fracture toughness of the material, respectively, and *β* = 0.15 and *d*_*c*_ are the critical cutting depths of the brittle–ductile transformation. If the cutting depth was greater than *d*_*c*_, the material would be removed in a brittle removal region, and if the cutting depth was less than *d*_*c*_, the material would be removed in a plastic removal region. As a consequence, for grinding, the removal mode of materials can be decided with the cutting depth of the grit.

In a study on the cutting depth of grit, Zhang et al. [28] proposed a new calculation method for determining the cutting depth of grit for the rotational grinding of workpieces, as shown in Equation (2). The values of various material property parameters required to calculate the critical cutting depth for the brittle–ductile transition of carbon fiber and glass fiber and the parameter values required to calculate the calculation method of the grit cutting depth are shown in Table 4 and Table 5, respectively.

(2) $$d_{g}=1.823r_{g}\sqrt[{3}]{\frac{fR_{1}n_{w}\tan\theta }{n_{s}^{2}\eta kW(D+W){\left(1+\sqrt{\frac{\pi H_{w}\tan\theta }{E_{t}^{*}}}\right)}^{2}}}$$

**Figure 11:**
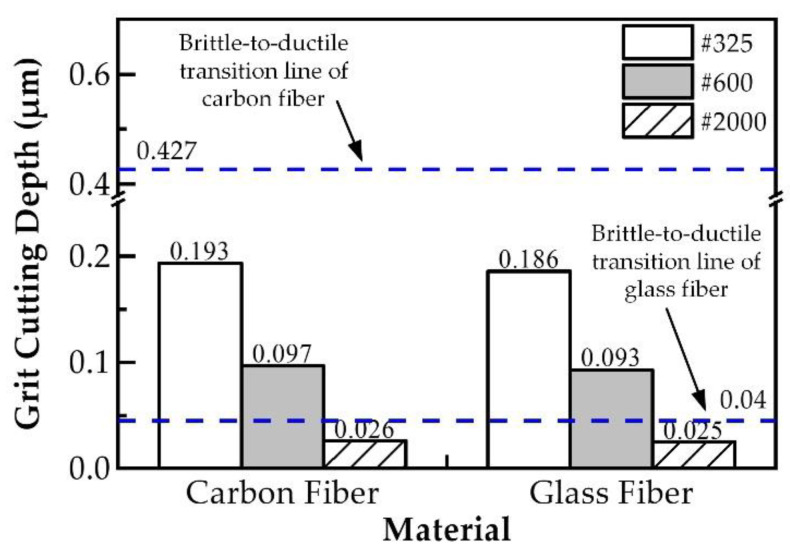
Comparing the cutting depth of the fiber brittle-to-ductile transition with the grit cutting depth of different grinding wheels.

The comparison results between the critical cutting depths of carbon fiber and glass fiber for the brittle–ductile transition and the grit cutting depths of carbon fiber and glass fiber in various grinding wheels are shown in Figure 11. It is know that the grit cutting depth is less than the brittle–ductile transformation depth of carbon fiber when the carbon fiber is ground with #325, #600 and #2000 wheels, which indicates that carbon fiber is removed in a ductile removal mode. When glass fibers were ground with the #325 and #600 wheels, the grit cutting depth was greater than the brittle–ductile transformation depth of the glass fibers, while the grit cutting depth was less than the brittle–ductile transformation depth of glass fibers when the glass fiber was ground with the #2000 wheels. This conclusion suggests that glass fibers were removed in a brittle removal mode when ground with the #325 and #600 wheels, and in a ductile removal mode when ground with the #2000 wheels.

The damage at the fiber–matrix interface was also one of the basic surface damages of the composite material. As can be seen from the surface morphology of the fiber-reinforced PEEK composites in Figure 7, Figure 8, Figure 9 and Figure 10, after grinding with #325 and #600 grinding wheels, fiber–matrix interface de-bonding was observed on either CF/PEEK or GF/PEEK surfaces, but after grinding with the #2000 grinding wheel, the interface de-bonding between the fiber and matrix obviously improved. During grinding, stress concentration may occur at the fiber–matrix interface. When stress exceeds the maximum bonding force provided by the fiber and matrix, the fiber–matrix interface can be damaged and de-bonding can occur. Due to the internal abrasive grit size of the #2000 grinding wheel being small, the radius of the grit was only 3.25 μm, and the number of grits per unit area of grinding wheel surface was large. As a result, the grinding force applied on individual grit was reduced, the grinding force at the fiber–matrix interface was also reduced and the grinding force provided by the individual grit did not exceed the bonding force between the fiber and matrix. This may be the reason why no obvious de-bonding phenomenon was observed on the material surface after grinding with the #2000 grinding wheel.

### 4.2. Surface Roughness Analysis for Grinding Processing

As shown in Figure 4a, the addition of carbon fibers to PEEK effectively reduced the surface roughness of the material, and the higher the fiber mass fraction, the lower the surface roughness. In general, the physical properties of the material affected the amount of the deformation of the surface material during grinding, and the material with high plasticity swelled more on both sides of the grooves than the material with weak plasticity. On the one hand, adding carbon fibers to PEEK could increase the crystallinity of the PEEK matrix [34], and as the crystallinity of PEEK increased, the tensile strength of PEEK and the modulus would increase and the plasticity capacity would weaken [35]. Therefore, adding carbon fibers could effectively reduce the degree of material swelling on both sides of the PEEK matrix grooves and improve the grinding surface quality. On the other hand, the CF30/PEEK’s nano-indentation curve was higher than CF10/PEEK [21], indicating that CF30/PEEK had a better deformation resistance. As seen in Figure 7 and Figure 8, the grooves on the surface of CF30/PEEK were shallower than those on CF10/PEEK after grinding with the same grit size grinding wheel.

The grooves on the grinding surface were formed by countless grits scratching the workpiece’s surface, and the average cut depth of grit was proportional to the material surface roughness after grinding. The value *k* in Equation (2) is the effective grit number that had a cutting effect on the surface layer of the grinding wheel in the grinding process, where the value *k* ranged from 0 to 0.5 [36]. The number of effective grits is influenced by the minimum chip thickness of the material, which is influenced by the ratio of the elastic modulus to the tensile strength of the material [37]. The larger the minimum cutting thickness of the material, the less effective grits can be cut on the surface layer of the grinding wheel.

It is worth noting that the value of *k* was empirical and could not be determined precisely; the general value for brittle materials is 0.2. However, both CF30/PEEK and GF30/PEEK were weak plastic materials. Thus, based on the tensile test results of Gao et al. [21], this paper sorted the minimum cutting thickness values of different materials by calculating the ratio of the elastic modulus to tensile strength and divided the value of *k* into five different values corresponding to five different materials in the test. Table 6 shows the value results.

**Figure 12:**
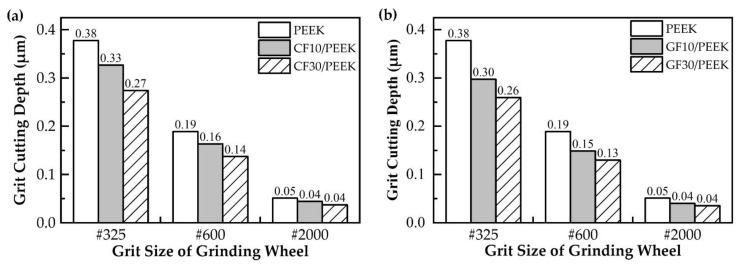
Comparison of grit cutting depth for different fiber-reinforced PEEK: (**a**) carbon-fiber-reinforced PEEK; (**b**) glass-fiber-reinforced PEEK.

The results of the grit cutting depth of different materials are shown in Figure 12. With the increase in the grit size of the mesh, the surface roughness was reduced due to the reduction in the grit cutting depth. In Figure 12a, CF30/PEEK had a smaller grit cutting depth than CF10/PEEK, so CF30/PEEK had less surface roughness than CF10/PEEK. In Figure 12b, although GF30/PEEK had a smaller grit cutting depth than GF10/PEEK, the glass fibers were removed in the brittle mode when grinding with the #325 and #600 wheels, and the broken glass fibers were distributed on the surface of the material, which deteriorated the surface quality, so GF30/PEEK had a larger surface roughness than GF10/PEEK.

## 5. Conclusions

Through the ultra-precision grinding experiment using PEEK, carbon-fiber- and glass-fiber-reinforced PEEK materials, this work explored the effects of reinforcing the fiber content and fiber type of the grinding machinability of fiber-reinforced PEEK composites. From the experimental results and discussion of the experimental results, the following conclusions could be drawn:

(1) Carbon fiber had a large brittle–ductile transition depth, which simplified the achievement of ductile removal in grinding. Additionally, the addition of carbon fibers enhanced the hardness of CF/PEEK, thus, improving the material’s resistance to deformation. As a result, the ground surface of CF/PEEK was better than that of pure PEEK under the same grinding conditions.

(2) With the increase in carbon fiber mass fraction, the ground surface roughness of CF/PEEK decreased due to the high deformation resistance during grinding. CF30/PEEK showed the best grinding machinability among all materials.

(3) The glass fiber brittle–ductile transition depth was small, but when the size of grit was large, the removal mode was easy to change to brittle removal, with broken glass fiber distributed on the surface of the material, reducing the surface quality. As a result, the surface roughness of GF/PEEK was highest among all materials when the removal mechanism of the glass fiber was brittle removal. When the size of the grit was small, the disparity between the ground surface roughness of GF/PEEK and CF/PEEK decreased.

## Figures and Tables

**Table 1:** Mechanical properties of materials used in this work [21,22].

Parameters	PEEK	CF10/PEEK	CF30/PEEK	GF10/PEEK	GF30/PEEK
Density (g/cm^3^)	1.31	1.34	1.40	1.38	1.51
Elastic modulus (MPa)	5100	7050	8190	5770	7330
Yield stress (MPa)	98.1	99.5	102.6	78.6	61.0
Hardness (MPa)	300	370	430	360	380
Elongation at break	20%	14%	7%	12%	5%

**Table 2:** Grinding experiment parameters for PEEK.

Grinding Condition	Value
Type of grinding wheel	Resin-bonded diamond grinding wheel
Grit size of grinding wheel	#325
Grinding wheel speed *n*_s_ (r/min)	2399, 2099, 1799
Wheel feed rate *f* (μm/min)	10, 15, 20
Rotary table speed *n*_w_ (r/min)	120, 180, 240
Material removal amount (μm)	100

**Table 3:** Grinding experiment parameters for fiber-reinforced PEEK.

Grinding Condition	Value
Type of grinding wheel	Resin-bonded diamond grinding wheel
Grit size of grinding wheel	#325, #600, #2000
Grinding wheel speed *n*_s_ (r/min)	2399
Wheel feed rate *f* (μm/min)	10
Rotary table speed *n*_w_ (r/min)	120
Material removal amount (μm)	100

**Table 4:** Carbon fiber and glass fiber material property parameters [25,29,30,31,32,33].

Parameters	Carbon Fiber	Glass Fiber
Modulus (GPa)	235	72.3
Hardness (GPa)	5.24	7.3
Fracture toughness (MPa·m^1/2^)	1.32	1.18

**Table 5:** Grit cutting depth model parameter values.

Parameters	Value
*r*_g_ (μm)	24 (#325), 12 (#600), 3.25 (#2000)
*R*_1_ (μm)	66,530
*n*_w_ (r/min)	120
*f* (μm/min)	10
*θ*	38°
*n*_s_ (r/min)	2399
*k*	0.2
*η*	0.25
*W* (μm)	3000
*D* (μm)	350,000
*E*_t_^*^ (GPa)	43.956

**Table 6:** *k* values for different materials.

Materials	*k* Value
PEEK	0.05
CF10/PEEK	0.075
CF30/PEEK	0.125
GF10/PEEK	0.1
GF30/PEEK	0.15

## Data Availability

The data presented in this study are available on request from the corresponding author.
